# Interactome of the oncogenic ΔNp73α isoform in human papillomavirus 38 E6/E7-transformed keratinocytes

**DOI:** 10.1128/mra.01071-24

**Published:** 2025-03-26

**Authors:** Luc Negroni, Valerio Taverniti, Massimo Tommasino, Katia Zanier

**Affiliations:** 1 Proteomics Platform, Institut de Génétique et de Biologie Moléculaire et Cellulaire (IGBMC)/INSERM U964/CNRS UMR 7104/Université de Strasbourg, Illkirch, France; 2 International Agency for Research on Cancer (IARC), World Health Organization, Lyon, Auvergne-Rhône-Alpes, France; 3 Biotechnology and Cell Signaling (CNRS/Université de Strasbourg, UMR 7242), Ecole Supérieure de Biotechnologie de Strasbourg 56848 , Illkirch, Grand Est, France; University of Guelph, Guelph, Ontario, Canada

**Keywords:** p53, deltaNp73, oncogenic viruses, HPV, interactome, AP-MS, cancer, proteomics, mass-spectrometry

## Abstract

ΔNp73α is a major oncogenic isoform of tumor suppressor p73. Here, we report ΔNp73α protein-binding partners in the organelle and cytoplasmic compartments of a cellular model consisting of human keratinocytes transformed by the E6 and E7 oncoproteins of the β-HPV38 virus associated with non-melanoma skin cancer.

## ANNOUNCEMENT

The tumor suppressor p53 family comprises three transcription factors (TFs), p53, p63, and p73, which, despite sharing high sequence identity, display specific functions and regulation. All three TFs are enriched in the nucleus, where they act on transcription and DNA repair, whereas their levels are regulated in the cytoplasm by ubiquitin-dependent or ubiquitin-independent degradation. In addition, mitochondrial localization has been reported for p53 and p73 (1). The genes coding for the three proteins (*TP53*, *TP63,* and *TP73*) can also be expressed as multiple isoforms (2). The highly carcinogenic ΔNp73α isoform investigated here is a dominant-negative inhibitor of full-length p53 and p73 (TAp53 and TAp73α) (3). It is generated by transcription from the internal P2 promoter of *TP73* and, consequently, lacks the N-terminal transactivation domain (2, 4, 5). Expression of viral oncoproteins can also result in ΔNp73α accumulation (6, 7), including the E6 and E7 oncoproteins from cutaneous beta (β) human papillomavirus (HPV) types (8–10) associated with non-melanoma skin cancer (11).

We have performed affinity purification-mass spectrometry (AP-MS) to characterize the ΔNp73α interactome using human keratinocytes immortalized by the β-HPV38 E6 and E7 oncoproteins as a model of cellular transformation (named 38HK hereafter) (8). To this end, we generated two stable cell lines, expressing either ΔNp73α fused to the N-terminus of the tandem affinity purification (TAP) tag (ΔNp73α-TAP) or TAP alone (negative control) (12). To discriminate between cytoplasmic versus nuclear or other organelle interactants, 38HK ΔNp73α-TAP and TAP 38HK cells were fractionated as described in our previous report (12). Briefly, 38HK cells were lysed mechanically in hypotonic buffer and centrifuged. The supernatant, corresponding to the cytoplasmic extract, was recovered. The pellet, containing nuclei and other organelles, was resuspended in buffer containing NaCl (250 mM) and glycerol (20%), incubated on ice, and centrifuged. The supernatant from this second centrifugation corresponds to the nuclear/organelle extract. Then, extracts were processed by tandem affinity purification (13) following the protocol described in Reference (12). Protein complexes were eluted from calmodulin beads and analyzed by SDS-PAGE and Western blotting or silver staining. Protein levels were quantified by the Bradford assay.

Eluates from calmodulin beads were precipitated with trichloroacetic acid, and the pellet was washed with acetone and dissolved with 2 M urea. Samples were digested with 100 ng of trypsin at 37°C for 10 h. The resulting peptide mixtures were directly analyzed by MS with a nanoLC U3000 in-line coupled with an Orbitrap Elite mass spectrometer using a nano-electrospray source (Thermo Scientific). MS measurements were performed on one biological sample per condition. Technical variability was estimated from three MS acquisitions for each sample (technical replicates). Peptides were separated using a C18 column (75 µm × 25 cm) with a 35 min linear gradient from 5% to 50% buffer B (A: 0.1% trifluoroacetic acid in H_2_O/B: 80% ACN, 0.08% trifluoroacetic acid in H_2_O). The mass spectrometer was operated in data-dependent mode with survey scans from m/z 300–1650 acquired in the Orbitrap at a resolution of 120,000 at m/z 400. Proteins were identified using Proteome Discoverer 2.5 software (Thermo Scientific), with false discovery rate (FDR) < 1% and at least one unique peptide. Label-free quantification was based on eXtracted Ion Chromatography (XIC) using the Minora node from Proteome Discoverer. Precursor and fragment mass tolerance were set at 7 ppm and 0.5 Da, respectively. Oxidation (M) was set as variable modification and carbamidomethylation (C) as fixed modification. The human fasta database with 20,419 sequences was obtained from Uniprot (https://www.uniprot.org/proteomes/UP000005640). Statistical analyses were performed using Perseus 1.6.1.5 according to the processing workflow described by the authors (14). The repeated *t*-test used for volcano plot analyses was based on standard parameters of Perseus (FDR 5%, S0 = 1).

**Fig 1:**
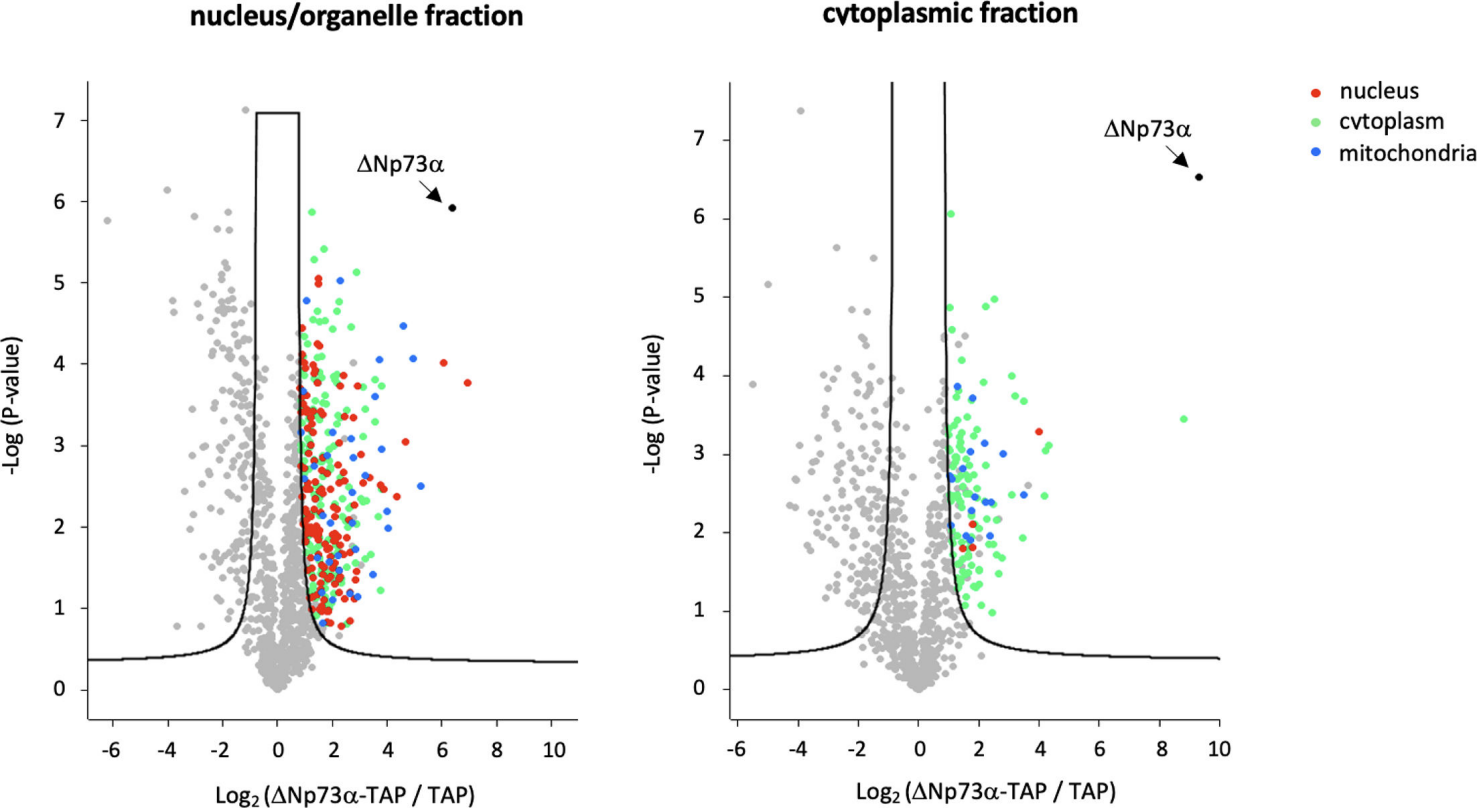
Volcano plot analysis of ΔNp73α partners in the nucleus/organelle (left) and cytoplasm (right) fractions of 38HK cells. The black continuous line indicates the limit of significance (FDR 5%, S0 = 1). Proteins situated on the right-hand side of this line (positive Log_2_ value) are significant interactants. These proteins are color coded according to the localization retrieved from the Gene Ontology Resource (https://geneontology.org). Red: nucleus; green: cytoplasm; blue: mitochondria; gray: localization not reported; black: ΔNp73α bait.

The lists of the top interactants of ΔNp73α (Log2FC > 2.1) in the nucleus/organelle and cytoplasmic fractions are reported in Table 1. The statistics from Perseus is presented in a volcano plot with each protein colored according to Gene Ontology Cellular Component (Fig. 1). The nuclear/organelle and cytoplasmic fractions are strongly enriched with protein partners with reported nuclear and cytoplasmic localization, respectively, thereby validating our biochemical approach. While more abundant in the nuclear/cytoplasmic fractions, mitochondrial binders are also present in the cytoplasmic fraction.

**TABLE 1:** Top interactants of ΔNp73α (Log2 FC >2.1)^*a*^^,^^*b*^

Log2 (FC)	-Log (P-value)	Accession	Gene name	Log2 (FC)	-Log (P-value)	Accession	Gene name
Nuclear/organelle fraction							
6.93	3.77	P09525	ANXA4	2.42	1.66	Q14257	RCN2
***6.4***	***5.92***	O15350	***ΔNp73α***	2.4	3.87	P28715	ERCC5
6.09	4.02	Q9H3D4	TP63	2.38	2.13	Q96IZ0	PAWR
5.26	2.51	P33121	ACSL1	2.38	2.35	Q13470	TNK1
4.97	4.06	P33121	MRPS22	2.33	1.36	P67775	PPP2CA
4.66	3.05	O95232	LUC7L3	2.33	1.68	Q8N9B5	JMY
4.58	4.48	P82673	MRPS35	2.32	1.31	Q9UI12	ATP6V1H
4.37	2.38	P50995	ANXA11	2.32	0.79	Q96RN5	MED15
4.03	1.98	Q92665	MRPS31	2.31	3.74	Q13573	SNW1
3.99	2.19	Q9Y2R5	MRPS17	2.3	3.34	O94915	FRYL
3.87	2.46	P27540	ARNT	2.3	1.4	Q9UHI6	DDX20
3.82	2.47	Q12965	MYO1E	2.28	5.03	Q6UB35	MTHFD1L
3.82	2.95	Q9Y3D9	MRPS23	2.28	1.78	Q14241	ELOA
3.8	3.74	O95425	SVIL	2.28	2.61	Q9UPN3	MACF1
3.78	2.51	O60828	PQBP1	2.27	1.36	O00443	PIK3C2A
3.77	1.22	Q96A65	EXOC4	2.26	4.77	P19013	KRT4
3.74	4.06	Q92552	MRPS27	2.26	1.2	Q58WW2	DCAF6
3.7	2.14	Q8IY21	DDX60	2.26	2.54	O15460	P4HA2
3.57	3.61	P82933	MRPS9	2.26	1.46	P82932	MRPS6
3.57	3.3	Q460N5	PARP14	2.25	1.47	Q9GZL7	WDR12
3.56	3.81	O43166	SIPA1L1	2.24	1.47	Q99959	PKP2
3.5	1.42	Q709C8	VPS13C	2.24	3.03	Q15427	SF3B4
3.41	1.67	Q9NP97	DYNLRB1	2.23	1.65	O43837	IDH3B
3.35	2.6	P62841	RPS15	2.22	1.7	Q9BU76	MMTAG2
3.27	2.32	O60573	EIF4E2	2.21	1.88	P56182	RRP1
3.22	3.45	Q9NYL9	TMOD3	2.21	3.37	Q96SU4	OSBPL9
3.22	1.61	Q9H788	SH2D4A	2.19	2.76	Q9UID3	VPS51
3.21	2.64	P82675	MRPS5	2.19	3.26	O75962	TRIO
3.14	2.31	Q07002	CDK18	2.19	3.77	Q5VSL9	STRIP1
3.14	2.55	O75676	RPS6KA4	2.19	2.22	Q8IXW5	RPAP2
3.12	3.12	P08758	ANXA5	2.18	4.65	Q86Y56	DNAAF5
3.12	3.72	Q9C037	TRIM4	2.18	3.88	Q96ED9	HOOK2
3.06	2.35	Q9BQS8	FYCO1	2.17	1.4	Q13144	EIF2B5
3.05	2.89	Q9BSJ2	TUBGCP2	2.16	1.67	Q99683	MAP3K5
3.04	1.53	A6NKD9	CCDC85C	2.12	1.78	Q15345	LRRC41
2.95	1.15	Q9Y3D3	MRPS16	2.11	3.81	Q14012	CAMK1
2.93	3.73	O00743	PPP6C	2.1	1.92	Q9NQG5	RPRD1B
2.89	5.13	P58107	EPPK1	2.1	2.29	Q15746	MYLK
2.88	1.46	P49841	GSK3B	2.1	1.83	P43686	PSMC4
2.87	2.04	Q6YN16	HSDL2	Cytoplasmic fraction			
2.87	1.36	Q7RTP6	MICAL3	***9.3***	***6.53***	***O15350***	***ΔNp73α***
2.85	1.73	P55084	HADHB	*8.8*	*3.45*	*Q9H3D4*	*TP63*
2.84	1.56	Q15628	TRADD	*4.31*	*3.12*	*O95816*	*BAG2*
2.83	2.27	P35244	RPA3	*4.22*	*3.05*	*P14735*	*IDE*
2.8	1.12	O95639	CPSF4	*4.18*	*2.47*	*Q9H4H8*	*FAM83D*
2.8	1.75	P15735	PHKG2	*4*	*3.29*	*O95232*	*LUC7L3*
2.79	2.86	P82930	MRPS34	*3.66*	*2.6*	*Q53RT3*	*ASPRV1*
2.78	4.02	Q9BY89	KIAA1671	*3.51*	*2.49*	*P33121*	*ACSL1*
2.77	3.34	Q8N5S9	CAMKK1	*3.49*	*3.67*	*Q9Y285*	*FARSA*
2.75	2.12	Q13395	TARBP1	*3.45*	*1.94*	*O14579*	*COPE*
2.74	2.43	P51398	DAP3	*3.19*	*3.75*	*Q7Z4S6*	*KIF21A*
2.73	2.05	O43615	TIMM44	*3.11*	*2.48*	*P01036*	*CST4*
2.73	1.77	Q9UHR6	ZNHIT2	*3.09*	*4*	*P53618*	*COPB1*
2.69	2.26	Q15404	RSU1	*2.82*	*3.01*	*Q8IVH4*	*MMAA*
2.69	3.09	P30038	ALDH4A1	*2.77*	*1.68*	*Q13200*	*PSMD2*
2.69	2.04	Q9UG63	ABCF2	*2.65*	*2.18*	*P12273*	*PIP*
2.69	4.46	Q08380	LGALS3BP	*2.65*	*1.48*	*P63098*	*PPP3R1*
2.69	4.45	O15296	ALOX15B	*2.58*	*2.16*	*Q6IQ49*	*SDE2*
2.67	1.69	Q9BVI4	NOC4L	*2.57*	*1.72*	*P36952*	*SERPINB5*
2.65	0.84	Q9Y6Q9	NCOA3	*2.52*	*4.98*	*Q9BPW8*	*NIPSNAP1*
2.64	1.19	P49411	TUFM	*2.47*	*2.37*	*Q9BYE4*	*SPRR2G*
2.64	1.19	Q9BQG0	MYBBP1A	*2.47*	*1.86*	*O94822*	*LTN1*
2.62	2.09	Q9UNQ2	DIMT1	*2.46*	*0.98*	*Q9C075*	*KRT23*
2.61	2.23	O95816	BAG2	*2.42*	*2.4*	*Q9BRT2*	*UQCC2*
2.6	2.5	P55209	NAP1L1	*2.36*	*1.96*	*P11310*	*ACADM*
2.58	2	Q99700	ATXN2	*2.32*	*1.86*	*Q99961*	*SH3GL1*
2.54	3.44	Q96II8	LRCH3	*2.27*	*2.18*	*P07858*	*CTSB*
2.53	1.51	Q13613	MTMR1	*2.24*	*2.86*	*P46199*	*MTIF2*
2.52	0.81	P46019	PHKA2	*2.23*	*4.89*	*P11142*	*HSPA8*
2.51	1.87	P78347	GTF2I	*2.21*	*2.38*	*P49590*	*HARS2*
2.5	2.08	Q5T5U3	ARHGAP21	*2.21*	*3.14*	*O75323*	*NIPSNAP2*
2.47	1.12	Q9NRA8	EIF4ENIF1	*2.18*	*2.41*	*P58107*	*EPPK1*
2.46	3.36	Q8WUM0	NUP133	*2.16*	*3.91*	*Q15418*	*RPS6KA1*
2.46	2.57	Q5GLZ8	HERC4				
2.46	3.09	Q86X10	RALGAPB				
2.43	2.68	O43148	RNMT				

^*a*^ All significant interactant at https://doi.org/10.5281/zenodo.14865996.

^*b*^ the ΔNp73α bait is indicated in bold/italics.

In our previous study (12), we have characterized the interaction of ΔNp73α with the E2F4/p130 transcriptional repressor complex, which is detected in the nuclear/organelle fraction of these analyses. Results show that E2F4 binds to ΔNp73α but not to the full-length TAp73α protein, suggesting that the interactomes of the oncogenic isoform and of the tumor suppressor protein may be different.

## Data Availability

The mass spectrometry proteomics data have been deposited to the ProteomeXchange Consortium via the PRIDE partner repository with the dataset identifier PXD056377. Lists of significant interactants (Log2 FC > 1) and relative statistics have been reported on the GitHub/Zenodo repository (https://doi.org/10.5281/zenodo.14865996).

## References

[1] Sayan AE, Sayan BS, Gogvadze V, Dinsdale D, Nyman U, Hansen TM, Zhivotovsky B, Cohen GM, Knight RA, Melino G. 2008. P73 and caspase-cleaved p73 fragments localize to mitochondria and augment TRAIL-induced apoptosis. Oncogene 27:4363–4372. doi:10.1038/onc.2008.64 18362891

[2] Murray-Zmijewski F, Lane DP, Bourdon J-C. 2006. P53/p63/p73 isoforms: an orchestra of isoforms to harmonise cell differentiation and response to stress. Cell Death Differ 13:962–972. doi:10.1038/sj.cdd.4401914 16601753

[3] Di C, Yang L, Zhang H, Ma X, Zhang X, Sun C, Li H, Xu S, An L, Li X, Bai Z. 2013. Mechanisms, function and clinical applications of DNp73. Cell Cycle 12:1861–1867. doi:10.4161/cc.24967 23708520 PMC3735700

[4] Lee CW, La Thangue NB. 1999. Promoter specificity and stability control of the p53-related protein p73. Oncogene 18:4171–4181. doi:10.1038/sj.onc.1202793 10435630

[5] Fontemaggi G, Kela I, Amariglio N, Rechavi G, Krishnamurthy J, Strano S, Sacchi A, Givol D, Blandino G. 2002. Identification of direct p73 target genes combining DNA microarray and chromatin immunoprecipitation analyses. J Biol Chem 277:43359–43368. doi:10.1074/jbc.M205573200 12213815

[6] Accardi R, Fathallah I, Gruffat H, Mariggiò G, Le Calvez-Kelm F, Voegele C, Bartosch B, Hernandez-Vargas H, McKay J, Sylla BS, Manet E, Tommasino M. 2013. Epstein - Barr virus transforming protein LMP-1 alters B cells gene expression by promoting accumulation of the oncoprotein ΔNp73α. PLoS Pathog 9:e1003186. doi:10.1371/journal.ppat.1003186 23516355 PMC3597522

[7] Allart S, Martin H, Detraves C, Terrasson J, Caput D, Davrinche C. 2002. Human cytomegalovirus induces drug resistance and alteration of programmed cell death by accumulation of deltaN-p73alpha. J Biol Chem 277:29063–29068. doi:10.1074/jbc.M201974200 12034725

[8] Accardi R, Dong W, Smet A, Cui R, Hautefeuille A, Gabet A-S, Sylla BS, Gissmann L, Hainaut P, Tommasino M. 2006. Skin human papillomavirus type 38 alters p53 functions by accumulation of deltaNp73. EMBO Rep 7:334–340. doi:10.1038/sj.embor.7400615 16397624 PMC1456898

[9] Cornet I, Bouvard V, Campo MS, Thomas M, Banks L, Gissmann L, Lamartine J, Sylla BS, Accardi R, Tommasino M. 2012. Comparative analysis of transforming properties of E6 and E7 from different beta human papillomavirus types. J Virol 86:2366–2370. doi:10.1128/JVI.06579-11 22171257 PMC3302372

[10] Accardi R, Scalise M, Gheit T, Hussain I, Yue J, Carreira C, Collino A, Indiveri C, Gissmann L, Sylla BS, Tommasino M. 2011. IkappaB kinase beta promotes cell survival by antagonizing p53 functions through DeltaNp73alpha phosphorylation and stabilization. Mol Cell Biol 31:2210–2226. doi:10.1128/MCB.00964-10 21482671 PMC3133237

[11] Rollison DE, Viarisio D, Amorrortu RP, Gheit T, Tommasino M. 2019. An emerging issue in oncogenic virology: the role of beta human papillomavirus types in the development of cutaneous squamous cell carcinoma. J Virol 93:e01003-18. doi:10.1128/JVI.01003-18 30700603 PMC6430537

[12] Taverniti V, Krynska H, Venuti A, Straub M-L, Sirand C, Lohmann E, Romero-Medina MC, Moro S, Robitaille A, Negroni L, Martinez-Zapien D, Masson M, Tommasino M, Zanier K. 2023. The E2F4/p130 repressor complex cooperates with oncogenic ΔNp73α to inhibit gene expression in human papillomavirus 38 E6/E7-transformed keratinocytes and in cancer cells. mSphere 8:e0005623. doi:10.1128/msphere.00056-23 36883841 PMC10117100

[13] Rigaut G, Shevchenko A, Rutz B, Wilm M, Mann M, Séraphin B. 1999. A generic protein purification method for protein complex characterization and proteome exploration. Nat Biotechnol 17:1030–1032. doi:10.1038/13732 10504710

[14] Tyanova S, Temu T, Sinitcyn P, Carlson A, Hein MY, Geiger T, Mann M, Cox J. 2016. The Perseus computational platform for comprehensive analysis of (prote)omics data. Nat Methods 13:731–740. doi:10.1038/nmeth.3901 27348712

